# The National Medical Union (Official)

**Published:** 1914-03-21

**Authors:** 


### THE NATIONAL MEDICAL UNION (Official).

Offices: 346 Strand. Tel.: City 8444.

HONORARY OFFICERS.

**Presidents.**

G. A. Wright, F.R.C.S. (Manchester).
Professor Wm. Russell (Edinburgh).

**Honorary Secretaries.**

Robert Carswell, M.A., M.B. (London).
E. Arthur Dorrell, F.R.C.S. (London).

**Vice-Presidents.**

Wm. Coates, C.B., D.L. (Manchester).
V. T. Greenyer, F.R.C.S. (Brighton).
John Playfair, M.D. (Edinburgh).
Bernard O'Connor, M.D., Barrister-at-Law (London).

**Honorary Treasurers.**

H. Oppenheimer, M.D., LL.D. (London), 19 College Crescent, Hampstead.
J. Brassey Brierley, M.D. (Manchester), 522 Stretford Road, Old Trafford.

**Provisional Council.**

J. Ford Anderson, M.D. (London).
J. L. Baskin, M.D. (Brighton).
A. Bousfield, M.D. (London).
W. F. R. Burgess, M.D. (London).
H. B. Greene, M.R.C.S. (London).
L. F. Hemmans, M.B., B.S., F.R.C.S.(Edin.) (London)
T. Johnston, M.R.C.S. (Brighton).
S. Lee, L.R.C.P.I. (London).
C. E. M. Lowe, M.B. (Crewe).
J. T. Macnamara, L.R.C.P.I. (London).
G. H. Pinder, M.R.C.S. (Manchester).
Frederick Porter, M.B. (Edinburgh).
J. Skardon Prowse, M.B. (Manchester).
Henry Sharman, M.D. (London).
A. F. Shoyer, M.D. (London).
James Smith, M.D. (Edinburgh).
F. W. Spurgin, M.R.C.S. (London).
T. J. Thyne, M.B., F.R.C.P. (Edinburgh).
F. H. Westmacott, F.R.C.S. (Manchester).
G. D. Wilson, L.R.C.P. (London).

### Formation.

The National Medical Union was formed at a Conference of Delegates from Non-Panel Associations, representing over 1,000 members, held in London on November 29, 1913. At this Conference it was unanimously resolved to form a Federation of Non-Panel Associations, of which the primary condition of membership should be that applicants for membership should not be on the panel directly or indirectly. The title "National Medical Union" was taken over, by consent, from the body of the same name formed at Manchester in 1912, which had taken the leading part in organising the Conference. The Union thus formed marks an important stage in the development of medical organisation. In relation to the Insurance Acts its attitude is one of absolute hostility; in this respect embodying the characteristics of its main sources of origin—namely, the National Medical Union of Manchester, the Edinburgh Medical Guild, and the London Medical Committee and Non-Panel Associations.

### The Manchester Union, formed at Manchester in 1912, with members all over the country, adopted at the very outset the uncompromising policy of "no service whatever" under the Insurance Act, a policy which it maintained throughout with a steadfastness in marked contrast to the unstable evolutions of the orthodox medical organisation then in possession of the field. In October 1913, acting on information supplied by the Wandsworth Non-Panel Association, the Manchester Union decided to summon the Conference which has resulted in the successful establishment of the new Union.

### The Edinburgh Medical Guild, a contemporary of the Manchester Union, has been characterised by its opposition to "contract practice," by its defence of the honourable ideals and traditions of the profession, and by the chivalrous way in which the leaders of the profession in Edinburgh have supported by their active participation the efforts of the general practitioners in defence of their freedom and principles.

### The London Medical Committee.

In London a remarkable fight was put up by the London Medical Committee, which organised the profession in the London area to such good purpose that after the lapse of a year of the working of the Act it was found that only 1,468 out of a total of 6,606 London practitioners could be cajoled or coerced into joining the panels, and that, too, in spite of special inducements in the shape of "secret" panels and two varieties of special contract forms being offered to them. Another gratifying result upon which the London Medical Committee may plume themselves is the large number of insured persons who have remained with their own doctors in preference to choosing one from the panel. It will thus be seen that London practitioners have played no small part in the non-panel movement.

### Objects.

The primary object of the Union is to organise the medical profession, in defence of its freedom and in protection of its economic interests, subject to the principal condition of membership—viz. that applicants shall not be on the panel directly or indirectly.

The dissatisfaction with the panel system so freely expressed by the insured public, and even by many who, until quite recently, were among its most ardent advocates; the inadequate and undermanned medical service; the deficit accumulating against the funds of the approved societies at the rate, so it is said, of twenty-seven shillings a minute; the shortage of funds to meet the demands of tuberculosis benefit, provide sanatoria, and pay the chemists; and, above all, the autocracy of an administration which has ignored the claims of insured persons to choose their own doctors, and at the same time alienated the larger and better half of the profession—all these developments point unmistakably to the conclusion that the machinery of the Insurance Act is breaking down. It is widely recognised that the interests of panel and non-panel practitioners are so opposed as to render it impossible for them to be represented in one and the same Association. Hence the National Medical Union has evolved, as the expression of a movement of cohesion on the part of those members of the medical profession who have, through thick and thin, adhered to the original attitude of opposition to which the medical profession as a whole at one time subscribed.

### Policy.

The Provisional Federating Council on the National Medical Union have formulated the following preliminary statement of objects and policy, viz.:—

1. To maintain the freedom of the medical profession in its professional and personal relations with the public.
2. To support the public in maintaining a corresponding freedom, and to safeguard the national health.
3. To oppose the present arrangements for medical benefit under the National Insurance Acts, and to decline professional service under such arrangements.
4. To preserve an independent attitude with regard to party politics.

To these four broad principles of policy will be added others of a more specific kind, upon which the Council is now busy with a view to recommending them for adoption at the first general meeting of the Union.

They will embrace such questions as the free choice of doctor, the disposal of the surplus medical benefit funds, the repeal of the medical benefit section of the Acts, or drastic amendment thereof, the question of voluntary as against compulsory insurance, and others designed to promote further organisation and concentration of non-panel efforts.

### Subscription.

The subscription to the Union is one guinea, payment of which entitles the subscriber to receive a weekly copy of The Hospital, in which is to be found notes recording the transactions of the Union.

---

### Proposed Local Medical Committee for London.

In reference to the resolutions circulated on the 12th inst. by the London Organisation Committee of the National Medical Union to all medical practitioners resident in the County of London and not on the panel, repudiating the action of the Metropolitan Counties Branch of the British Medical Association in attempting to elect a Local Medical Committee for London, the hon. secretaries have been instructed to publish the following information:—

The proposals of the Metropolitan Counties Branch are made with a view to the Committee so to be elected obtaining statutory recognition by the Insurance Commissioners as "representative of duly qualified medical practitioners resident in the county," such Committee when elected and recognised to have the duties mentioned in Section 62 of the Insurance Act of 1911.

Section 62, 1911, reads as follows:—

"Where a local medical committee has been formed for any county or county borough, and the Insurance Commissioners are satisfied that such committee is *representative of the duly qualified medical practitioners resident in the county*, they shall recognise such committee, and, where a local medical committee has been so recognised, it shall, subject to the regulations made by the Insurance Commissioners, be consulted by the Insurance Committee on all general questions affecting the administration of medical benefit, including the arrangements made with medical practitioners giving attendance and treatment to insured persons, and shall perform such other duties and shall exercise such powers as may be determined by the Insurance Commissioners."

The Amending Act, Section 32, of 1913, provides that the opinions and wishes of the medical practitioners who have entered into the agreements shall be ascertained by the Insurance Committees through Panel Committees representative of the panel practitioners. Such Panel Committee has been formed in London, and it is to be noted that by the Amending Act it is made eligible for recognition as the Local Medical Committee above described in Section 62 of the principal Act.

---

The London Organisation Committee of the National Medical Union bases the recommendations announced in the circular on two grounds:

1. The numerical basis of the election proposed by the Metropolitan Counties Branch renders it quite unrepresentative of the whole profession resident in the County of London, and on that ground it would be ineligible for statutory recognition, and illegal if recognised.
2. The duties and sphere of action of the Panel Committee and Statutory Medical Committee respectively in London being as above described, and the situation with regard to panel and non-panel practitioners in London being what it is, the London Organisation Committee of the National Medical Union regards the attempt by the Metropolitan Counties Branch to set up a Statutory Committee on the above lines as calculated to involve the non-panel practitioners in London in an appearance of lending support to the present arrangements for medical benefit under the Insurance Acts, whereas, as a matter of fact, their aim and object is to proclaim and evince in every possible way their determined opposition to the same.

**For the Institutional Worker Supplement see pages following p. xlii.**

